# Quantitative evaluation and selection of reference genes in mouse oocytes and embryos cultured in vivo and in vitro

**DOI:** 10.1186/1471-213X-7-14

**Published:** 2007-03-06

**Authors:** Solomon Mamo, Arpad Baji Gal, Szilard Bodo, Andras Dinnyes

**Affiliations:** 1 Genetic Reprogramming Group, Agricultural Biotechnology Center, Szent Gyorgyi Albert ut 4, H-2100 Gödöllő, Hungary; 2 Research Group for Applied Animal Genetics and Biotechnology, Hungarian Academy of Sciences and Szent Istvan University, H-2100 Gödöllő, Hungary

## Abstract

**Background:**

Real-time PCR is an efficient tool to measure transcripts and provide valuable quantitative information on gene expression of preimplantation stage embryos. Finding valid reference genes for normalization is essential to interpret the real-time PCR results accurately, and understand the biological dynamics during early development. The use of reference genes also known as housekeeping genes is the most widely applied approach. However, the different genes are not systematically compared, and as a result there is no uniformity between studies in selecting the reference gene. The goals of this study were to compare a wide selection of the most commonly used housekeeping genes in mouse oocytes and preimplantation stage embryos produced under different culture conditions, and select the best stable genes for normalization of gene expression data.

**Results:**

Quantitative real time PCR method was used to evaluate 12 commonly used housekeeping genes (*Actb, Gapdh, H2afz, Hprt, Ppia, Ubc, Eef1e1, Tubb4, Hist2h2aa1, Tbp, Bmp7, Polr2a*) in multiple individual embryos representing six different developmental stages. The results were analysed, and stable genes were selected using the geNorm software. The expression pattern was almost similar despite differences in the culture system; however, the transcript levels were affected by culture conditions. The genes have showed various stabilities, and have been ranked accordingly.

**Conclusion:**

Compared to earlier studies with similar objectives, we used a unique approach in analysing larger number of genes, comparing embryo samples derived in vivo or in vitro, analysing the expression in the early and late maternal to zygote transition periods separately, and using multiple individual embryos. Based on detailed quantification, pattern analyses and using the geNorm application, we found *Ppia, H2afz *and *Hprt1 *genes to be the most stable across the different stages and culture conditions, while *Actb*, the classical housekeeping gene, showed the least stability. We recommend the use of the geometric averages of those three genes for normalization in mouse preimplantation-stage gene expression studies.

## Background

Preimplantation embryo development is a dynamic developmental process recognized by four distinct phases [1] that vary in stage and duration from species to species (reviewed in [2]). These phases span the time after fertilization until the formation of blastocyst, and further differentiation to the inner cell mass (ICM) and trophectoderm. During preimplantation stage embryo development, the expression of some active transcripts peculiar to each stage has been described earlier [3-5]. The different developmental stages are marked with variations in the cell number, total and poly (A) RNA contents [6-8]. Understanding such biological dynamics during early embryonic development would yield insights into the complex molecular pathways controlling early development [9], and further refinement of assisted reproductive technology (ART) in mammals [10].

Common methods of RNA detection and analyses were described elsewhere [11,12]. However, technical limitations and dearth of starting material have restricted accurate, quantitative analysis of mRNA abundance for genes of interest in mammalian oocytes and early embryos, using the classical molecular biology approaches [12-14]. Real time PCR has been a quantitative method of choice to understand the comparative roles of different transcripts in the preimplantation-stages of embryo development, and to corroborate the results of microarray and other gene expression studies. It has greatly improved the quantitative gene expression studies, due to its speed, ease of use, reproducibility, high sensitivity, and absence of radioactive materials [11]. The values of real time PCR quantitative results, besides good experimental and primer designs, lie in the accurate applications of all the procedures like quality RNA isolation, cDNA synthesis, dilutions made, pippeting, use of appropriate controls, and final analysis [15]. Moreover, embryonic samples have additional sources of variations. Unlike cell lines and single-organ tissues, the cells comprising the embryo have inherently a vastly heterogeneous nature, which leads to greater variation in the endogenous biological processes, and greater variation in the sensitivity of the cells to the treatment [16]. There is high probability that any of these factors can introduce intra- and inter assay variations for which normalization is required.

Besides standardizing most of these procedures to control variations, different normalization procedures were used so far. The pros and cons of different normalization approaches were described in a recent review [17]. Internal reference genes, which are also known as housekeeping genes, are used in most experiments to normalize the results of gene expressions, albeit variations in selecting the type of gene. Different studies have used the most commonly known reference genes that include β-actin (*Actb*), glyceraldehydes-3-phosphate dehydrogenase (*Gapdh*), hypoxantin-guanine phosphoribosyl transferase (*Hprt1*), and 18S ribosomal RNA [17,18]. Owing to the pattern variations of these genes under different conditions, their unconditional uses were frequently criticized [18-20]. A number of studies started to address the issue by evaluating normalizer genes for different species, including ovine [21], and bovine [13,14,22]. In a recent mouse study [12], the results were based on comparisons of only a few genes and developmental stages.

In the present study, the goals were to compare the expression of a wider selection of the most commonly used housekeeping genes (12 genes) in mouse oocytes and different preimplantation-stage embryos and finally select the best stable genes for normalization. To our knowledge, for the first time, comparisons of the early and late phases of the maternal to zygotic development control transitions (MZT), and embryos derived both in vivo or produced in vitro (IVP) have been made in the same study, to make the results more widely applicable.

## Results

### Primer screening and PCR efficiency analyses

For all primers, optimisation runs were performed prior to initial screening and quantitation experiments. The minus RT reaction and design of most primers at the exon-exon junction enabled us to control absence of contaminating genomic DNA. Owing to the uniformity of the initial primer design criterion used, it was possible to use similar reaction conditions for all the primers during real time PCR assays. In the screening analysis, similar cDNA dilutions from the pooled embryos were used, and the absence of dimers and C_T _values were compared. The C_T _is defined as the number of cycles needed for the fluorescence to reach a specific threshold level of detection and is inversely correlated with the amount of template nucleic acid present in the reaction [23]. All the selected genes were detected throughout the preimplantation development stages but with various signal intensities as observed from the C_T _values. Using similar low concentration template for all, seven genes were detected at C_T _values below 35 and the rest five genes were detected at C_T _values closer to 40. Recognizing the scarcity of embryo materials at preimplantation stages, the seven genes with earliest signals (earlier C_T_values) were preferred and selected for further comparisons (Table 1).

**Table 1:** Reference genes selected for the study, and sizes of the PCR products.

Symbol	Gene name	Product size (bp)	Reference Sequence	Locus link
Actb*	Actin, beta, cytoplasmic	192	NM_007393	11461
Gapdh*	Glyceraldehydes-3-phosphate dehydrogenase	98	NM_001001303	407972
Hprt1*	Hypoxanthine guanine phosphoribosyl transferase	117	NM_013556	15452
H2afz*	H2A histone family, member Z	202	NM_016750	51788
Ubc*	Ubiquitin	112	NM_019639	22190
Ppia*	Peptidylprolyl isomerase A	150	NM_008907	268373
Eef1e1*	Eukaryotic translation elongation factor 1 epsilon 1	110	NM_025380	66143
Tubb4	Tubulin, beta 4	167	NM_009451	22153
Hist2h2aa1	Histone 2, H2aa1	182	NM_013549	15267
Tbp	TATA box binding protein	122	NM_013684	21374
Bmp7	Bone morphogenetic protein 7	145	NM_007557	12162
Polr2a	Polymerase (RNA) II (DNA directed)	139	NM_009089	20020

* Genes selected after the initial screening and used for the subsequent comparisons.

### Gene expression profile analyses at different developmental stages

**Figure 1:**
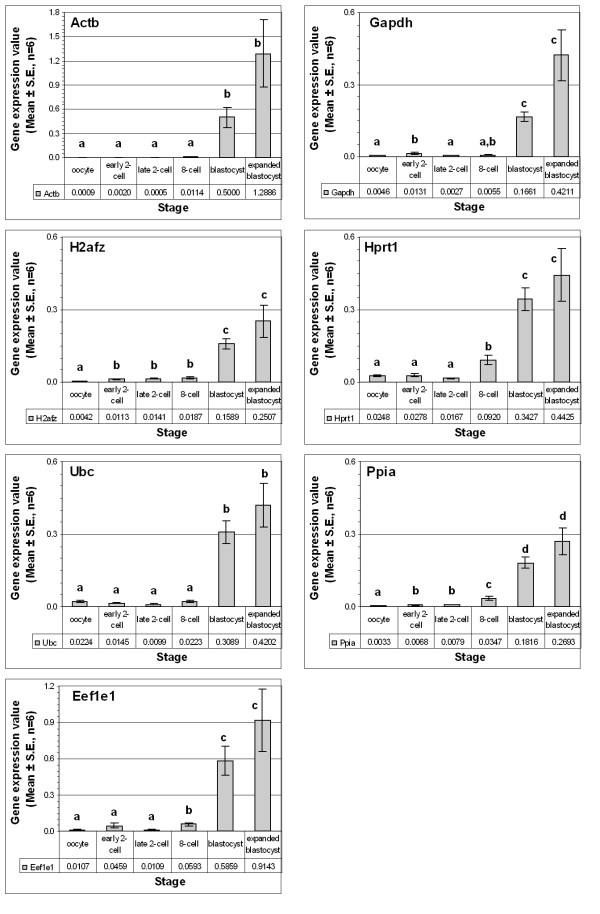
Individual expression profiles of selected reference genes in the in vivo derived embryos. The expression level in a particular developmental stage was represented with in vitro produced blastocyst embryo equivalent values, to show the relative amount. Stages with different letters are significantly (P ≤ 0.05) different for the expression of the gene.

**Figure 2:**
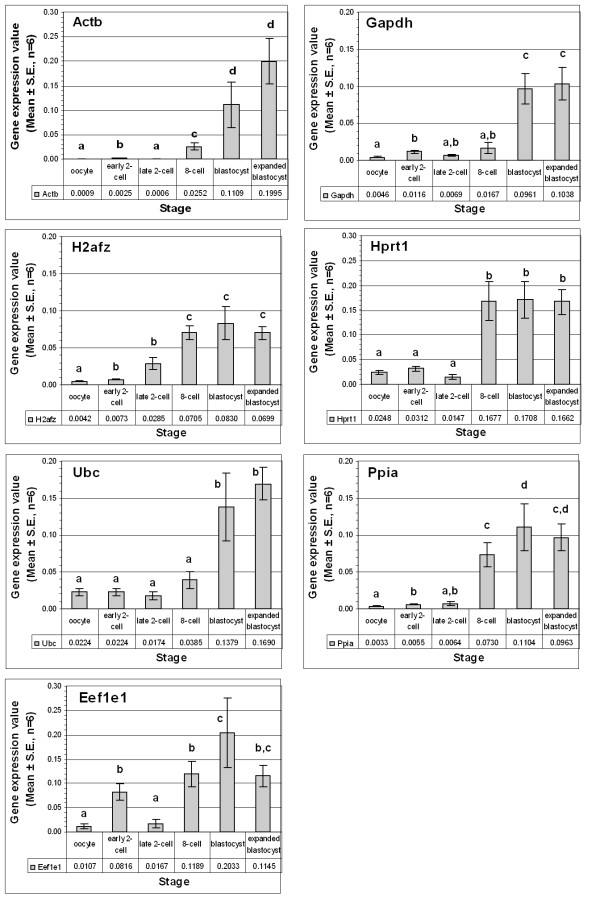
Individual expression profiles of selected reference genes in the in vitro produced embryos. The expression level in a particular developmental stage was represented with in vitro produced blastocyst embryo equivalent values, to show the relative amount. Stages with different letters are significantly (P ≤ 0.05) different for the expression of the gene.

For the selected seven housekeeping genes, transcript quantifications from multiple individual embryo cDNA preparations (6 individuals for each stage and each culture condition) were done under identical experimental procedures. Generally, the expression of almost all of them surged at the beginning of the first cell cycle (2-cell stage). Except the observed moderate changes for the transcripts of *Ubc *and *Hprt1*, the transcripts for the rest of the genes increased by at least 70% at this stage. In both in vivo (Figure 1) and in vitro produced (Figure 2) samples, the highest increase at the early 2-cell stage was observed for the *Eef1e1 *(eukaryotic translation elongation factor 1, epsilon 1) gene. However, starting from the late 2-cell stage, the pattern varied among the genes. The expression of the genes *H2afz *and *Ppia *exceptionally increased concomitant with advancement of the developmental stages. For the rest of the genes, the surge at the early 2-cell stage was immediately followed by a transient reduction at the late 2-cell stage and increased continuously thereafter. Despite the difference in the culture conditions, the above expression patterns remained generally the same (compare Figure 1 and Figure 2). However, the levels varied according to the embryo source as described in the next section.

### Gene expression profile analyses in different culture conditions

**Figure 3:**
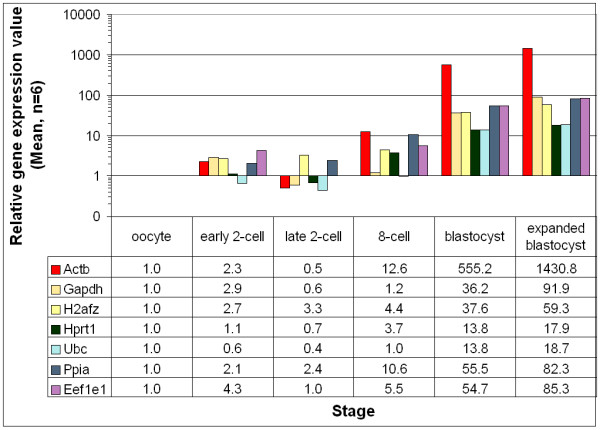
Relative expression levels of different transcripts in the in vivo derived preimplantation-stage mouse embryos. The expression at the oocyte stage was taken as a reference to calculate the relative amounts in the different stages.

**Figure 4:**
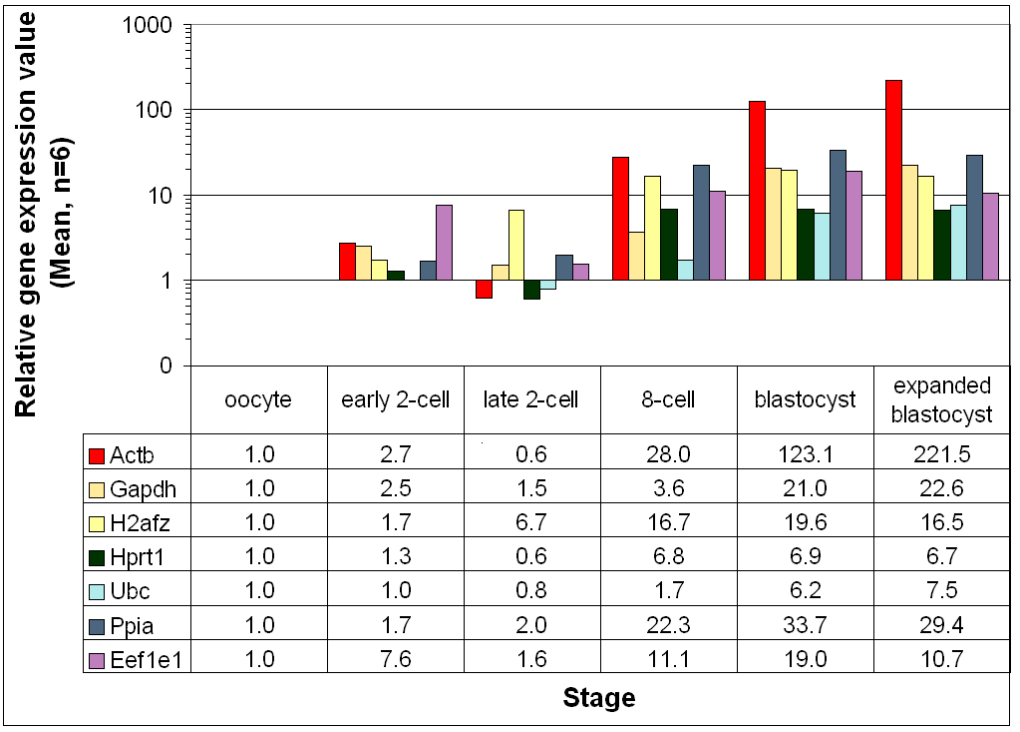
Relative expression levels of different transcripts in the in vitro produced preimplantation-stage mouse embryos. The expression at the oocyte stage was taken as a reference to calculate the relative amounts in the different stages.

Expression profiles of the selected housekeeping genes at different developmental stages were compared in the in vivo derived vs. IVP embryo samples. Despite the variations of embryo sources (in vivo and in vitro), the temporal expression patterns remained similar. But the stage-by-stage comparisons revealed differential transcript levels between the samples of the two embryo culture sources. Taking the expression of a gene at the oocyte-stage as a reference, for the same developmental stages and sample volume, relatively more transcript copy numbers were observed for the in vitro samples until the 8cell stage. However, except the significant variations (P ≤ 0.05) for the histone (H2afz) at the early 2-cell and 8-cell stage, the rest were not significant (P > 0.05). These patterns were dramatically changed at the early and hatched blastocyst stages with much higher transcript copy numbers for the in vivo samples (Figure 3) compared to the in vitro ones (Figure 4), and the variations at these later stages were significantly different (P ≤ 0.05) for all the genes (compare Figure 3 and Figure 4).

### Gene expression stability analysis

**Figure 5:**
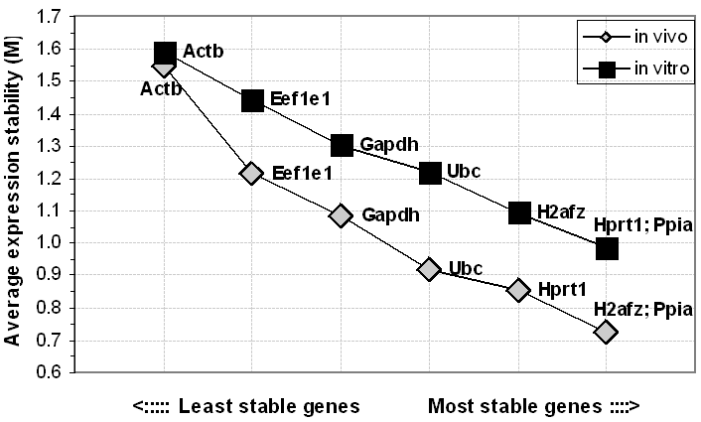
Average gene expression stability values of the selected reference genes in different cultures as calculated by geNorm software and ranking made based on the relative stability values.

The gene expression stability analysis over the different embryonic stages and culture conditions, using geNorm software, ranked the genes based on their stability measure value (M) calculated. Accordingly, the genes *Ppia, H2afz *and *Hprt1 *were found to be the most stable, followed by the genes *Ubc, Gapdh, Eef1e1 *and *Actb *in their order of appearance. *Actb *is the least stable gene observed with the highest M value in both in vivo and IVP embryos (Figure 5). Generally, the differences in the culture conditions have had little effect on the order of stability for the majority of them. However, the stability measure value (M) of a particular gene is higher in the in vitro samples compared to the value for the same gene in the in vivo samples (Figure 5).

## Discussion

Recognizing the variations of the dynamics in gene expression of different tissues [11], developmental stages [24], and treatment conditions [25,26], the identification of stable normalizer genes for each experimental condition was frequently suggested [17,27,28]. The use of unconfirmed reference genes for normalization is misleading the interpretation of the gene expression results [29,30]. As a result, RNA mass quantity was frequently used for normalization. However, this approach has been challenged for not considering the variations during the subsequent enzymatic reactions, its impracticality for normalizing mRNA transcripts and small samples like microdissected tissues and embryos [31]. On the other hand, the competitive PCR procedure of adding exogenous template, although used by others [32,33], has also been criticized for its laborious procedures [34,12], and competition with endogenous sequences for primers and nucleotides during the PCR reactions [13]. Moreover, the method is not accounting for the quality and quantity of the input RNA [22], although the reference gene expression stability can be affected by RNA quality [35]. The use of housekeeping genes for normalization is a widely used approach in most experiments, and a number of publications appeared to suggest housekeeping genes for different experimental conditions.

Recently, two groups [12,36] published their finding, on appropriate housekeeping genes for mouse oocytes and preimplantation-stage embryos. Despite the valuable contributions of the Jeong et al. [12] in examining the effects of different RNA isolation and detection techniques on the selection of reference genes, the recommendations on the reference gene aspect was constrained by the limited number of genes compared, developmental stages and culture systems considered. Taking into consideration the broad options (for selections) of reference genes, and the different embryo production systems used for various experiments, it is imperative to consider wider options to come to meaningful recommendations. Although the later work [36] tried to address the issue of reference genes, the aspect on the embryo was constrained by its design to consider advanced peri-implantation and post-implantation stage embryos only (days 3.5, 7.5, 9.5 and 11.5), which is not a representative of full preimplantation developmental period. Moreover, two of the selected genes in our experiment (Ppia and H2afz) were not considered, and the use of pooled samples is another difference from our experiment.

Our approach to select the appropriate housekeeping genes focused on the use of multiple individual embryos, as compared to pooled samples. The results of various previous studies [37,38] have supported the use of individual samples as compared to the pooled ones in depicting the true biological variations. Moreover, to comply with the scarcity of materials in the embryo samples, advantages of early signal detection and strong correlation coefficients were leading to the selection of seven genes for further quantification and evaluations. Comparison of these genes can make our recommendations applicable to use even in studies of rare transcripts. Thus, we believe that our approach has enabled to identify the most stable housekeeping genes for normalization in the systems examined.

One of the interesting observations in this study was the transcript variation between the early and late 2-cell stage embryos. In most studies so far, 2-cell stage was taken in a holistic analysis without due regard for the different time courses. In mouse embryo development, the 2-cell stage is a bridge from the maternal phase to the embryonic phase of development control (maternal-zygotic transition, MZT), and marked by a lag in the development (developmental block). Thus, compared to other stages, the fractional analysis of transcripts at this stage is important, as it enables to identify the most stable genes for normalization even under major transcript shifts during development. The observations in this study with variations in transcript levels among the early and late 2-cell stage embryos support this concept.

The effects of culture conditions on the temporal gene expression patterns of preimplantation-stage embryo development in mouse have been described earlier [39-42]. Studies with other genes demonstrated the effects of culture conditions on the expression patterns of genes [43-46]. As far as we know, the comparisons of gene expression profiles of housekeeping genes under different culture conditions have not been made simultaneously earlier. In the current study, in vitro samples showed higher copy numbers until the 8-cell stage, and lower copy number at the blastocyst stages compared to the in vivo samples at the same developmental stages. To our knowledge such dynamics of stage-specific variations between the in vivo and in vitro samples were not detected in the earlier housekeeping genes studies. The transcript variations within the same developmental stage and cell number (until 8-cell stage), although not significant for most genes, can only be related to the difference in the culture conditions. The significant (P ≤ 0.05) variations in the transcript copy number at the blastocyst stages can be partially attributed to the larger cell numbers of in vivo blastocysts compared to the in vitro blastocysts (39 ± 8.1 vs. 32 ± 8.6). But the variations cannot be explained only with cell number, and thus, needs further investigation. The results of the analyses have finally enabled us to select the most stable reference genes to be considered for normalization of gene expression results in mouse preimplantation-stage embryos. The relatively stable expression of the three genes (*Ppia, H2afz *and *Hprt1*) throughout the different preimplantation-stages was further verified at the late 2-cell stage, when the transcripts of most other genes were repressed, yet these genes showed a stable expression in both culture conditions. Even with a narrow selection of genes, an earlier mouse study [12] has also suggested the use of *H2afz *gene for normalizations. Despite the established trends of using a single reference gene for normalization, the approach has frequently been criticized. The error related to using a single reference gene has been clearly indicated, and the authors [31] proposed to calculate the normalization factor (NF) based on the use of a geometric mean of at least three housekeeping genes, carefully selected for expression stability. Thus, we recommend the use of the geometric averages of the above three genes for mouse preimplantation-stage gene expression result normalizations. However, the number of genes used for geometric averaging is a trade-off between practical considerations and accuracy [31]. For example, in our recent work where the Hprt-deficient mouse strain was used for the experiment, we used the averages of the rest two genes and the results were biologically sound (unpublished data). The frequently used *Actb *gene was the worst performing gene and our study revealed this fact by making the gene stability analysis for all the genes considered in this study (Fig 3). In a previous study by our group [47], variations in Actb levels following vitrification of 8-cell stage embryos by one of the cryopreservation methods (probably with more adverse effects than in other groups) created difficulties in comparing different methods and interpreting the results. Earlier studies in mouse [48,12], and bovine [24,22] have also observed similar variations for the *Actb *gene. Although the selected constitutive genes were not much different between the two culture systems, the minor variations in the order of the genes (Table 2) can be attributed to the observed variations in the transcript levels between the two cultures.

**Table 2:** Primer sequences and parameters of standard curves for the selected genes used in the experiment

Gene	Forward Reverse	Slope (m)	Intercept (b)	R^2^
Actb	ATGAGCTGCCTGACGGCCAGGTCATC TGGTACCACCAGACAGCACTGTGTTG	-2.97	22.16	0.99
Gapdh	TGACGTGCCGCCTGGAGAAA AGTGTAGCCCAAGATGCCCTTCAG	-3.49	21.69	0.98
Hprt1	GCTTGCTGGTGAAAAGGACCTCTCGAAG CCCTGAAGTACTCATTATAGTCAAGGGCAT	-3.57	23.02	0.97
H2afz	ACAGCGCAGCCATCCTGGAGTA TTCCCGATCAGCGATTTGTGGA	-3.81	21.20	0.99
Ubc	CGTCGAGCCCAGTGTTACCACCAAGAAGG CCCCCATCACACCCAAGAACAAGCACAAG	-3.67	23.32	0.97
Ppia	CGCGTCTCCTTCGAGCTGTTTG TGTAAAGTCACCACCCTGGCACAT	-3.71	20.84	0.99
Eef1e1	GCGGAGTTGAGGCTGCTGGAGA AGACTCGGGCCATTGTTTGTCTG	-2.96	26.95	0.96

## Conclusion

The effects of culture conditions and developmental stages on the expression of the genes were studied in detail for a wide selection of reference genes and compared both under in vivo and in vitro culture systems. Our result shows that it is possible to use the same selected genes for both culture systems, however culture conditions affected the transcript levels. Therefore, calculation of different normalization factors, which is sample specific, is necessary.

The stable expression of the three reference genes (*Ppia, H2afz *and *Hprt1*) concomitant with the advancing developmental stage warrants their selection as normalizer for mouse preimplantation stage embryo gene expression analysis. The least stability observed for β-actin in both culture conditions, imply its inappropriateness as reference gene. Results of the current study and those in other mammalian species revealed the need for system specific reference genes. Although the selected reference genes were evaluated under in vivo, and our in vitro culture conditions (CZB-Hepes), we suggest further evaluation under various in vitro culture (KSOM, SOF, M16, etc) conditions.

## Methods

All chemicals, unless stated otherwise, were purchased from Sigma-Aldrich Chemical Inc. (St. Louis, USA).

### Oocyte collection

Female ICR (CD1) mice, aged 7 to 8 weeks old, were induced to superovulate by intraperitoneal administration of 5.0 IU pregnant mare serum gonadotropin (PMSG, Folligon^® ^Intervet, The Netherlands), and then 48 h later, by 5.0 IU of human chorionic gonadotropin (hCG, Choregon^®^, Richter Gedeon Rt., Hungary). Donor female mice were humanly killed at 16 h post hCG injection and cumulus oocyte complexes were collected from the ampullae of the oviducts with subsequent removal of the cumulus cells using hyaluronidase (1 mg/ml) in CZB-Hepes buffer. Seven oocytes were individually collected for mRNA isolation. Finally, the matured oocytes were washed three times in RNAse-free water, collected individually in 2 μl drops of RNase-free water and stored at -80°C until RNA extraction.

### Embryo production and culture conditions

Female ICR mice, aged 7 to 8 weeks old, were induced to superovulate as described earlier. Each injected female was mated with a single, more than 10 weeks old male of the same strain, which was subsequently verified by the presence of vaginal plug. Female mice were humanly killed, at specific times for each developmental stage, and in vivo samples of early (38 h) and late (46 h) 2-cell stage embryos, 8-cell stage embryos (65 h), early (93 h) and expanded blastocysts (93 h) were collected.

For the IVP samples of the same developmental stages, pronuclear zygotes were flushed by opening the ampullae at 20 hr post-hCG administration and, the cumulus cells were removed using hyaluronidase in CZB-Hepes buffer. The zygotes were then selected based on the presence of two pronuclei and cultured in a group of 20 in CZB medium as described earlier [47], until the proper developmental stage [early 2-cell (38 h), late 2-cell (47 h), 8-cell (87 h), early blastocyst (94 h) and expanded blastocyst (111 h)].

Finally, the different culture source and developmental-stage embryos were washed three times in RNAse-free water, collected individually in 2-μl drops of RNase-free water and stored at -80°C until RNA extraction.

### RNA isolation and cDNA synthesis

The procedures of RNA isolation and cDNA synthesis were as described in our earlier works [15,49]. Briefly, messenger RNA was isolated individually from 6 embryos per developmental stage and culture condition using Dynabeads^® ^mRNA DIRECT™ Micro Kit (Dynal A.S, Oslo, Norway), following the manufacturer's instructions. The individually frozen embryos were lysed and incubated with pre-washed magnetic Dynabeads that can base pair with poly (A) tails of mRNA molecules. After hybridisation and subsequent repeated washes with buffers, the RNA was eluted in RNase-free water and reverse transcribed into cDNA, using M-MLV RT kit (Invitrogen, Carlsbad, CA) in a final 20-μl reaction volume. Minus RT reaction was performed to check the absence of contaminating residual DNA.

### Primer design and real time PCR analysis

A total of twelve genes, most commonly used as housekeeping for normalization, were selected for evaluation throughout the different developmental stages and culture conditions. Primers were designed for these genes at the exon-exon border using Primer Express Software (Applied Biosystems, Foster City, CA), optimised and initially screened using similar concentration templates. Details of the primers are described in Table 1 and Table 2.

The details of real time PCR reaction procedures were as described earlier [49]. During quantification of the transcripts, the assay for each gene consisted of six replicates per stage, six different preimplantation embryo developmental stages, negative and positive controls. All genes were compared from the same stock to avoid inter-assay template variations. Each sample in a run consisted of 0.08 embryo equivalent cDNA template, 300 nM of each primer, and 50% SYBR^® ^Green JumpStart™ Taq ReadyMix™ in 25-μl reaction volume. The reaction conditions were template denaturation and polymerase activation at 95°C for 2 min followed by 45 cycles of 95°C denaturation for 15 sec, 60°C annealing for 20 sec and 72°C extension for 30 sec. The reactions were carried out using the Rotor-Gene™ 3000 real time PCR machine (Corbett Research, Mortlake, Australia), and the results were analysed with the integrated Rotor-Gene software (version 6.0.27). At the end of PCR reactions, melt curve analyses were performed for all genes, and the specificity as well as integrity of the PCR products were confirmed by the presence of a single peak. For the selected genes, the expected sizes of the products were also confirmed by gel electrophoresis on a 2% agarose gel stained with ethidium bromide and visualized under UV light. For calculating PCR efficiencies, standard curves were generated from assays made with serial dilutions of cDNA preparations using 5-pooled blastocysts. Moreover, to ensure the comparability of PCR assays, three independent serial dilutions were made that enabled us to determine the C_T _values and PCR efficiencies of the individual assay, and calculate the correlation between them. PCR efficiency (E) was calculated with the equation (E = (10^[-1/slope] ^- 1) × 100).

### geNorm and expression stability analysis

Analysis of the gene expression stability over the different embryonic stages was performed using the geNorm software [31]. The analysis relies on the principle that the expression ratio of two ideal internal control genes is identical in all samples, regardless of the experimental condition or cell type, and determined as the standard deviation of the logarithmically transformed expression ratios [31]. Using the software, the internal control gene stability measure value (M) was calculated as the average pair wise variation of a particular gene with respect to the rest of the genes, and ranking was made based on these values. The lower the M value, the more stable the expression of the gene under consideration. The most stable reference genes were identified by stepwise exclusions of the least stable gene and recalculating the M values.

## Authors' contributions

SM conceived the experiment, performed the experimental design, all the molecular genetic analysis, interpretation of data and was the primary author of the manuscript. ABG participated in the experimental design, performed primer design, data analysis and statistical tests, and also participated in manuscript preparation. SB prepared in vivo and in vitro embryos for the experiment and participated in manuscript preparation. AD participated in experimental design, supervised the study, helped to draft the manuscript and approved the final version. All authors read and approved the manuscript.
